# The Roles of Coenzyme A Binding Pocket Residues in Short and Medium Chain Acyl-CoA Synthetases

**DOI:** 10.3390/life13081643

**Published:** 2023-07-28

**Authors:** Yu Meng, Cheryl Ingram-Smith, Oly Ahmed, Kerry Smith

**Affiliations:** 1 Department of Genetics and Biochemistry, Clemson University, Clemson, SC 29634, USA; ymeng@kean.edu (Y.M.); oahmed@g.clemson.edu (O.A.); 2 College of Science and Technology, Wenzhou-Kean University, Wenzhou 325060, China

**Keywords:** acetyl-CoA, coenzyme A, acetyl-CoA synthetase, acyl-CoA synthetase, methanoarchaea

## Abstract

Short- and medium-chain acyl-CoA synthetases catalyze similar two-step reactions in which acyl substrate and ATP bind to form an enzyme-bound acyl-adenylate, then CoA binds for formation of the acyl-CoA product. We investigated the roles of active site residues in CoA binding in acetyl-CoA synthetase (Acs) and a medium-chain acyl-CoA synthetase (Macs) that uses 2-methylbutyryl-CoA. Three highly conserved residues, Arg^193^, Arg^528^, and Arg^586^ of *Methanothermobacter thermautotrophicus* Acs (Acs_Mt_), are predicted to form important interactions with the 5′- and 3′-phosphate groups of CoA. Kinetic characterization of Acs_Mt_ variants altered at each of these positions indicates these Arg residues play a critical role in CoA binding and catalysis. The predicted CoA binding site of *Methanosarcina acetivorans* Macs (Macs_Ma_) is structurally more closely related to that of 4-chlorobenzoate:coenzyme A ligase (CBAL) than Acs. Alteration of Macs_Ma_ residues Tyr^460^, Arg^490^, Tyr^525^, and Tyr^527^, which correspond to CoA binding pocket residues in CBAL, strongly affected CoA binding and catalysis without substantially affecting acyl-adenylate formation. Both enzymes discriminate between 3′-dephospho-CoA and CoA, indicating interaction between the enzyme and the 3′-phosphate group is important. Alteration of Macs_Ma_ residues Lys^461^ and Lys^519^, located at positions equivalent to Acs_Mt_ Arg^528^ and Arg^586^, respectively, had only a moderate effect on CoA binding and catalysis. Overall, our results indicate the active site architecture in Acs_Mt_ and Macs_Ma_ differs even though these enzymes catalyze mechanistically similar reactions. The significance of this study is that we have delineated the active site architecture with respect to CoA binding and catalysis in this important enzyme superfamily.

## 1. Introduction

Acetyl-CoA synthetase (Acs) plays fundamental roles in the metabolism and physiology of cells from all three domains of life [1,2], and its regulation by acetylation is well studied [3]. Acs and other short and medium chain acyl-CoA synthetases catalyze a two-step reaction in which the first step Equation (1) requires acyl substrate and ATP but not CoA for formation of an enzyme-bound acyl-AMP intermediate with release of inorganic pyrophosphate (PP_i_) as a product. In the second step Equation (2), the acyl group is transferred to the sulfhydryl group of CoA and the acyl-CoA and AMP products are released.

E + acyl substrate + ATP ⇆ E•acyl-AMP + PP_i_ (1)

E•acyl-AMP + HSCoA ⇆ E + acyl-CoA + AMP (2)

Structures of Acs from *Saccharomyces cerevisiae* (Acs_Sc_; PDB ID 1RY2) and *Salmonella enterica* (Acs_Se_; PDB ID 2P2F) [4,5] represent conformations of the enzyme in the acyl-adenylate-forming Equation (1) and thioester-forming Equation (2) steps, respectively. These structures indicate that the C-terminal domain rotates 140° toward the N-terminal domain in the transition between the two steps of the reaction. This domain alternation has been proposed to form the complete active site for proper positioning of CoA for nucleophilic attack on the acyl group of the intermediate during catalysis of the second half-reaction Equation (2) [5,6].

The 2.1 Å crystal structure of Macs_Ma_ (PDB ID 3ETC), a medium chain acyl-CoA synthetase from *Methanosarcina acetivorans* [7], revealed that in the absence of substrate this enzyme is in a similar conformation to that for thioester formation. This was surprising, given that the Acs_Se_ structure in this same conformation was obtained from enzyme crystallized in the presence of adenosine 5′propylphosphate, which mimics the acetyl-adenylate intermediate and CoA [5]. Recently, the structure of the *Lathyrus sativus* oxalyl-CoA synthetase was solved in the presence of ATP and oxalate but not CoA and was also found to adopt the thioester forming conformation [8].

Our characterization of the *Methanothermobacter thermautotrophicus* Acs (Acs_Mt_) and *Archaeoglobus fulgidus* Acs (Acs_Af_) revealed that these enzymes are more diverse in substrate utilization than previously thought [9]. Whereas the acyl substrate range for Acs_Mt_ is limited to acetate and propionate with a strong preference for acetate, Acs_Af_ has a broader acyl substrate range that includes butyrate, valerate, and the branched-chain isobutyrate, and has only a slight preference for acetate over propionate. The *Pyrobaculum aerophilum* Acs likewise has an expanded acyl substrate range [10].

Characterization of Macs_Ma_ revealed that the preferred acyl substrate is the branched chain 2-methylbutyrate [11]. The enzyme has a broad acyl substrate range for the acyl-adenylate forming step of the reaction, with the ability to utilize propionate (C_3_) to octanoate (C_8_) as well as certain branched chain substrates; however, the acyl-adenylate formed with many of these substrates was not suitable for the thioester-forming second step of the reaction and was released in the absence of CoA. CoA inhibited acyl-AMP release and instead promoted its breakdown to AMP and the acyl group, which were released along with PP_i_ [11]. In the presence of 2-methylbutyrate, Macs_Ma_ did not release the acyl-AMP intermediate in the absence of CoA and in the presence of CoA completed the two-step reaction and released 2-methylbutyryl-CoA, AMP, and PP_i_ as products [11].

As Acs and Macs catalyze similar two-step reactions that differ only in the acyl substrate, it was expected that these enzymes would have similar active site architecture in which the acyl substrate binding pocket is expanded to accommodate larger substrates. We have shown that Trp^416^ in Acs_Mt_ (Trp^414^ in Acs_Se_) plays an essential role in determining acyl substrate range and preference [12]. This Trp in almost completely conserved among Acs sequences but is replaced by Gly in medium chain acyl-CoA synthetases. Based on our results, other labs have engineered the acyl substrate pocket of Acs to utilize novel substrates to generate alternative acyl-CoA substrates for metabolic engineering [13,14,15].

Inspection of the Acs_Se_ and Macs_Ma_ crystal structures [5,7] and our analysis of site-directed variants altered in the acyl substrate pocket of Macs_Ma_ and Acs_Mt_ [11,12] indicate fundamental differences in the active site architecture of the two enzymes. Trp^416^ of Acs_Mt_ is replaced by Gly in Macs_Ma_, as would be expected, and an alternate Trp residue, Trp^259^, occupies a position similar to that of Trp^416^ and was shown to be critical for substrate binding and catalysis [11,12].

ATP binding site determinants have been investigated in Acs [16,17] but not Macs. However, signature motif III (YXXGD) of the acyl-adenylate-forming enzyme superfamily [18], shown by Ingram-Smith et al. [16] to play a key role in ATP binding and catalysis in Acs, is well conserved in Macs_Ma_ as ^431^YHTGD^435^. The Asp at the last position in motif III is invariant among superfamily members and interacts with one or both hydroxyl groups of the ribose moiety of ATP in all of the structures available thus far, including that of Macs_Ma_ [7], suggesting that residues in this motif may serve similar roles in ATP binding in both Acs and Macs.

Short- and medium-chain acyl-CoA synthetases are widespread in the archaea [9] and have provided a rich background for studying the structural and biochemical diversity within this family. Here we report our investigation of the CoA binding sites of Macs_Ma_ and Acs_Mt_. As previously shown for acyl substrate binding and catalysis of the first step of the reaction, our results indicate that key residues involved in CoA binding and catalysis of the second step of the reaction in Acs_Mt_ are dispensable in Macs_Ma_. Instead the CoA binding site of Macs_Ma_ more closely resembles that of 4-chlorobenzoate CoA ligase (CBAL), which catalyzes the formation of 4-chlorobenzoyl-CoA [19,20,21,22,23].

## 2. Materials and Methods

### 2.1. Site-Directed Mutagenesis

Site-directed alteration of the Macs_Ma_ and Acs_Mt_ gene was accomplished with the QuickChange kit (Stratagene, cat. 200519) and the altered sequences were confirmed by sequencing. Oligonucleotides for site-directed mutagenesis were purchased from Integrated DNA Technologies (www.idtdna.com).

### 2.2. Purification of Macs_Ma_ and Acs_Mt_ Enzymes

The Macs_Ma_ and Acs_Mt_ enzymes were heterologously produced in *Escherichia coli* Rosetta Blue (DE3) *placI* (EMD Millipore) as described previously [11,12]. Clarified cell lysate was applied to a 5 mL His-Trap column and purified protein was eluted using a linear gradient of increasing imidazole concentration in buffer. The purified enzymes were dialyzed against 25 mM Tris, 10% glycerol [pH 7.5], aliquoted, and stored at −20 °C. Protein concentrations were determined by the Bradford method [24] using Bio-Rad Protein Assay Kit II (Bio-Rad, cat. 5000002) according to the manufacturer’s instructions.

### 2.3. Assay for Acyl-CoA and Acyl-Adenylate Production

The hydroxamate assay [25,26] measures production of activated acyl groups, including both acyl-CoA and acyl-adenylate. Reaction mixtures (0.3 mL containing 100 mM Tris-HCl [pH 7.5] (Fisher Scientific, cat. BP152-5) and 600 mM hydroxylamine-HCl (Acros, cat. 270100010) [pH 7.0]) with varied concentrations of acyl substrate, MgATP (Fisher Scientific, cat. BP413-25), and CoA (Fisher Scientific, cat. BP25101). Reactions were stopped by the addition of two volumes (0.6 mL) stop solution [1 N HCl, 5% trichloroacetic acid (Acros, cat. 152130010), 1.25% FeCl_3_ (Fisher Scientific, I88-500)]. The change in absorbance at 540 nm was measured and product formation was calculated by comparison to a standard curve. Reactions were performed at the optimal temperature for each enzyme (55 °C for Macs_Ma_ and 65 °C for Acs_Mt_). For ethanol-soluble acyl substrates, the concentration of the stock solutions were adjusted such that the final ethanol concentration in the reaction was kept constant at 2%. All reactions were performed in triplicate.

For determination of apparent kinetic parameters, the concentration of each substrate was varied individually while the concentrations of the other substrates were held constant at a saturating level (~5–10 times the *K_m_* for that substrate). The apparent kinetic parameters with their standard errors were calculated using non-linear regression to fit the data to the Michaelis-Menten equation. All reactions were performed in triplicate. Values are the mean ± standard deviation.

### 2.4. Assay for Inorganic Pyrophosphate Production

Pyrophosphate production by Macs_Ma_ was determined by the molybdate assay as described in Meng et al. [11]. Briefly, reactions (0.3 mL) were performed at 55 °C and reaction mixtures contained 50 mM Tris-HCl [pH 7.5], 4 mM MgCl_2_, 10 mM dithiothreitol (Fisher Scientific, cat. BP172-25). The concentrations of ATP, CoA, and acyl substrate were varied for determination of kinetic parameters. Reactions were terminated at 10 min by addition of 0.08 mL H_2_O, 0.05 mL 0.5 M 2-mercaptoethanol (Fisher Scientific, cat. O34461-100), 0.05 mL molybdate reagent [2.5% ammonium molybdate (Fisher Scientific, cat. A674-500) in 5 N H_2_SO_4_], and 0.02 mL Eikonogen reagent [25 mM sodium sulfite (Fisher Scientific, cat. S447-500), 13 mM 1-amino-2-naphthol-4-sulfonic acid (Themo Scientific, cat. B24339-22), 963 mM sodium meta-bisulfite (Fisher Scientific S244-500)]. The absorbance at 580 nm was measured after 10 min and compared to a PP_i_ standard curve. All reactions were performed in triplicate. Values are the mean ± standard deviation.

### 2.5. Assay for Acyl-CoA Thioester Bond Formation

Acyl-CoA thioester bond formation was measured as previously described [27]. Briefly, reactions (0.5 mL) were performed at 55 °C in 100 mM Tris-HCl (pH 7.5) with a range of substrate concentrations. Acyl-CoA thioester bond formation was measured spectroscopically at 233 nm. All reactions were performed in triplicate. Values are the mean ± standard deviation.

## 3. Results

### 3.1. Conserved Arg Residues in Acs Interact with CoA

**Figure 1:**
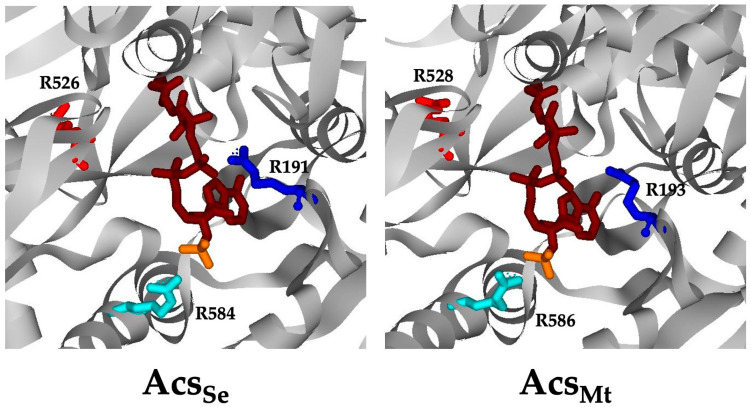
CoA binding region of AcsSe and AcsMt. The Acs_Mt_ structure (**right**) was modeled on AcsSe (**left**; PDB ID 2P2F). CoA is shown in magenta, with the 3′-phosphate group in orange. Corresponding Arg residues in each structure (Acs_Se_/Acs_Mt_) are displayed as follows: Arg^526/528^ in red, Arg^191/193^ in blue, and Arg^584/586^ in aqua.

Inspection of the Acs_Se_ structure reveals interaction between the negatively charged phosphate groups of CoA and two conserved Arg residues, Arg^191^ and Arg^584^, with Arg^191^ interacting with both the 5′-diphosphate and 3′-phosphate groups and Arg ^584^ interacting with just the 3′-phosphate of CoA [5]. An additional highly conserved Arg residue, Arg^526^, interacts with the phosphate group of the acyl-adenylate intermediate and has been predicted to play a role in stabilizing the thioester-forming conformation [5]. These three Arg residues are conserved in Acs_Mt_ as Arg^193^, Arg^528^, and Arg^586^, respectively, and occupy similar positions relative to CoA (Figure 1).

Each of these Arg residues was individually altered to Ala, Lys, and Gln in Acs_Mt_ and kinetic parameters were determined for the purified enzyme variants. Overall, alterations at Arg^193^ had the most severe effect on the *K_m_* value for CoA. The *K_m_* values for CoA for the Arg^193^Lys and Arg^193^Gln variants increased 18.9- and 41.0-fold, respectively, and the Arg^193^ Ala variant was unsaturable for CoA (Table 1). The Arg^586^ and the Arg^528^ variants generally showed much less of an effect on the *K_m_* for CoA, with increases ranging from less than two-fold up to 8.8-fold except for the Arg^528^Ala variant, which was rendered unsaturable for CoA (Table 1).

The turnover rates for all the Arg variants were significantly impaired (Table 1), with 34- to 38-fold reductions in *k*_cat_ observed for the Arg^586^Lys and Arg^586^Ala variants, 160- to 291-fold reductions for the Arg^193^Lys and Arg^193^Gln variants, and 130- to 326-fold reductions for the Arg^528^Gln and Arg^528^Lys variants. The most severe reduction in catalysis was observed for the Arg^586^Gln variant, which displayed a 680-fold reduced *k*_cat_. The effects on the overall catalytic efficiency with CoA ranged from a 58-fold reduction for the Arg^586^Lys variant to a nearly 12,000-fold reduction for the Arg^193^Gln variant. Even the more conservative Arg^193^Lys alteration resulted in ~3000-fold reduced catalytic efficiency, suggesting Arg^193^ plays a critical role in catalysis as well as CoA binding.

The *K_m_* values for ATP and acetate were also determined for each variant. Alterations at the targeted Arg residues had only minor effects on the *K_m_* for ATP (Supplemental Table S1). The *K_m_* for acetate for most of the variants was similar to that for the wild-type enzyme with the exception of the Arg^193^Ala, Arg^528^Ala, and Arg^586^Gln variants which were unsaturable for acetate even at concentrations as high as 800 mM (Supplemental Table S1).

### 3.2. Interaction between Arg^586^ and the 3′-Phosphate Group of CoA Is Important for Substrate Binding and Catalysis

Based on the Acs_Se_ structure, Arg^586^ of Acs_Mt_ is predicted to interact with the 3′ phosphate group of CoA. To examine the contribution and nature of this interaction in CoA binding and catalysis, we examined whether the unaltered enzyme and the Arg^586^Ala and Arg^586^Lys variants could discriminate between CoA and 3′-dephospho CoA. The wild-type enzyme had over 10-fold higher *K_m_* for 3′-dephospho CoA than for CoA but catalysis was not greatly reduced. The resulting 26.5-fold higher catalytic efficiency with CoA versus 3′-dephospho CoA (Table 2) indicates that the interaction between the enzyme and the 3′-phosphate group plays an important role in CoA binding.

The Arg^586^Ala variant had a 6-fold higher *K_m_* value for CoA but similar *K_m_* value for 3′-dephospho CoA as the wild-type enzyme. Catalysis was greatly reduced with either substrate, resulting in just 2.2-fold difference in catalytic efficiency with CoA versus 3′-dephospho CoA (Table 2), indicating this variant can no longer discriminate well between the presence and absence of the 3′-phosphate group. Retention of a positive charge at position 586 in the Arg^586^Lys variant was not sufficient to restore discrimination between CoA and 3′-dephospho CoA. The *K_m_* for CoA was less than 2-fold increased versus that of the wild-type enzyme. This variant had a lower *K_m_* for 3′-dephospho CoA than the wild-type enzyme or the Arg^586^Ala variant, but *k*_cat_ was still greatly reduced resulting in only 3.7-fold preference for CoA versus 3′-dephospho CoA (Table 2).

### 3.3. Electrostatic Interaction between Macs_Ma_ and the 3′-Phosphate Group of CoA Is Important

**Figure 2:**
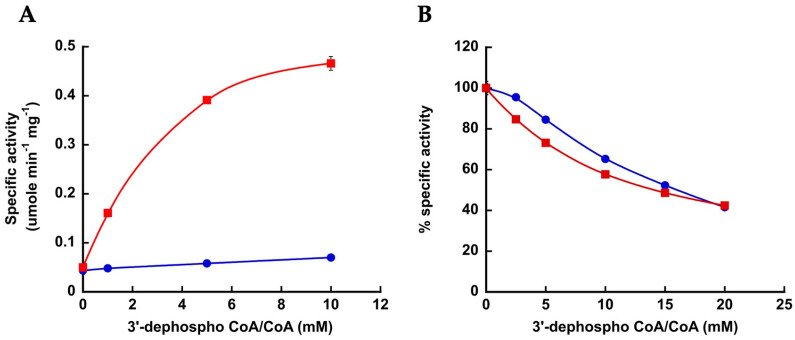
Effect of CoA and 3′-dephospho CoA on the acyl-CoA synthetase and propionyl-adenylate synthetase activities of Macs_Ma_. (**A**) Acyl-CoA synthetase activity of Macs_Ma_ with CoA or 3′-dephospho CoA. Activity was measured at increasing concentrations of either CoA (red) or 3’-dephospho CoA (blue). Specific activities shown are the mean ± standard deviation of three replicates. (**B**) Inhibition of the propionyl-CoA synthetase activity of Macs_Ma_ by CoA (red) versus 3′-dephospho CoA (blue). Activities shown are the percent activity measured in the absence of CoA (100%) versus presence of CoA and are the mean ± standard deviation of three replicates.

To examine whether Macs_Ma_ also makes an electrostatic interaction with the 3′-phosphate group of CoA, the ability of wild-type enzyme to discriminate between CoA and 3′-dephospho CoA was determined. The enzyme displayed very low 2-methylbutyryl-CoA synthetase activity with 3′-dephospho CoA even at a concentration of 10 mM, whereas the activity observed with 10 mM CoA was over 5-fold higher (Figure 2A). Kinetic parameters could not be determined with 3′-dephospho CoA, so the level of discrimination could not be ascertained.

In the absence of CoA, wild-type Macs_Ma_ catalyzes synthesis and release of an acyl-adenylate when less favorable acyl substrates such as propionate are used, and the presence of CoA inhibits this activity [11]. Inhibition of the acyl-adenylate synthetase activity by CoA versus 3′-dephospho CoA was examined as another means for determining whether interaction between the enzyme and the 3′-phosphate group of CoA is important. The acyl-adenylate synthetase activity was inhibited by both CoA and 3′-dephospho CoA to a similar extent (Figure 2B), suggesting that interaction with the 3′-phosphate group is important for CoA binding for the second step of the reaction but does not play a role in interaction between CoA and the enzyme for the first step of the reaction or when the second step cannot occur.

### 3.4. The CoA Binding Pocket in Macs_Ma_ Resembles That in CBAL

The CoA nucleotide binding pocket in the Acs_Se_ and CBAL structures differs but the pantetheine tunnel is similar [5,20]. In CBAL, the aromatic residues Phe^473^ and Trp^440^ play key roles in CoA binding and catalysis by accommodating the adenine moiety of CoA. Alterations of these residues greatly reduced catalytic efficiency for the second step of the reaction while having little effect on first step [22]. Arg^475^ interacts with the CoA 3’-phosphate [5,20] and alteration reduced catalytic efficiency [22].

**Figure 3:**
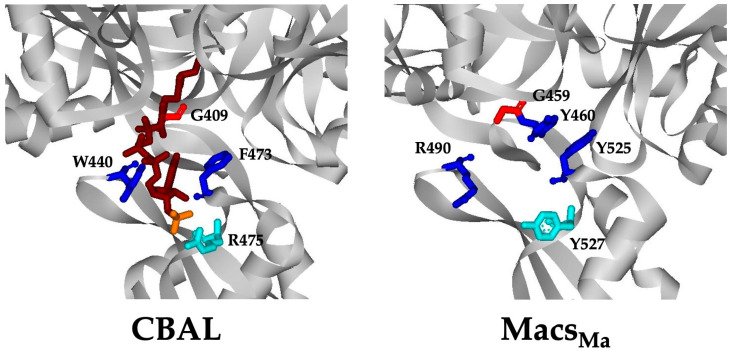
The CoA binding region of CBAL and Macs_Ma_. The CBAL structure (**left**; PDB ID 3CW9) has CoA bound (in magenta with the 3′-phosphate group in orange). Residues shown to play an important role in CoA binding and catalysis are indicated. Residues predicted to be important for CoA binding in Macs_Ma_ (**right**; PDB ID 3ETC) are shown in the same color as the corresponding residues in CBAL.

Comparison of the Macs_Ma_, Acs_Se_, and CBAL structures revealed that the CoA binding site of Macs_Ma_ more closely resembles that of CBAL [21]. In Macs_Ma_, Tyr^525^ and Arg^490^ replace Phe^473^ and Trp^440^ of CBAL, respectively (Figure 3), although Tyr^460^ of Macs_Ma_ is also positioned such that it could function similarly to Trp^440^ of CBAL, which interacts with the adenine moiety of CoA [5,20]. Tyr^527^ of Macs_Ma_ occupies a similar location to Arg^475^ of CBAL but the side chain is positioned away from the 3′-phosphate and may instead interact with the ribose group of CoA via its benzoyl group [21]. Gly^459^, located in close proximity to the putative CoA binding site of Macs_Ma_, is highly conserved among all members of the adenylate-forming enzyme superfamily and has been proposed to be necessary to open the pantetheine tunnel in the thioester-forming conformation [21].

Based on these structural comparisons, we investigated the role of Macs_Ma_ residues Gly^459^, Tyr^525^, Tyr^460^, Arg^490^ and Tyr^527^ in CoA binding and catalysis. Alterations were made at each of these residues and the recombinant enzyme variants were produced and purified. The Tyr^460^Ala and Arg^490^Ala variants were insoluble and were not characterized. Kinetic parameters were determined for the purified enzyme variants to examine the impact of the alterations on acyl-CoA synthetase activity. Alteration of Gly^459^ to Ala had little effect on enzymatic activity. The *K_m_* and *k*_cat_ values showed just slight changes from those for the wild-type enzyme for the 2-methylbutyryl-CoA synthetase activity (Table 3 and Supplemental Table S2).

**Figure 4:**
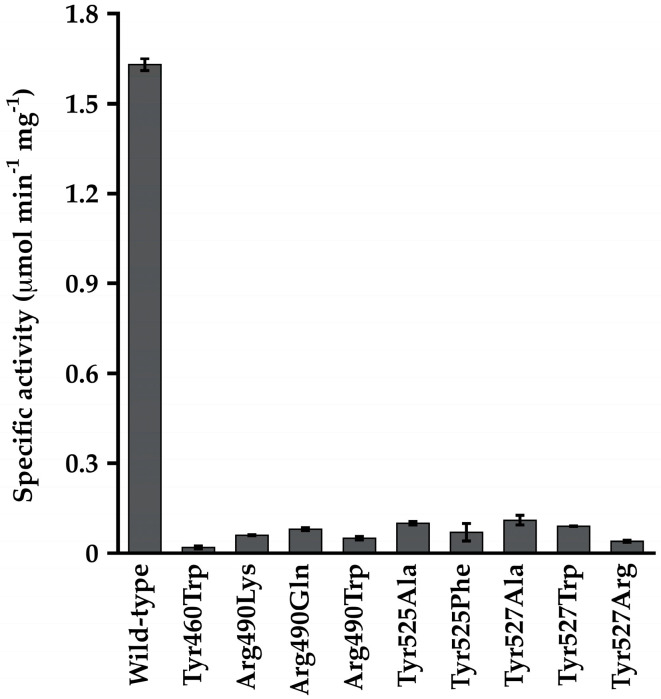
2-methylbutyryl-CoA synthetase specific activity of wild-type Macs_Ma_ and the Tyr^460^, Arg^490^, Tyr^525^, and Tyr^527^ variants determined in the presence of 15 mM CoA.

Alterations at Tyr^460^, Tyr^525^, Tyr^527^, and Arg^490^ proved to be very deleterious to the acyl-CoA synthetase activity of Macs_Ma_, with little 2-methylbutyryl-CoA synthetase activity observed even at high CoA concentrations. These variants displayed 15- to 80-fold reduced specific activity relative to the wild-type enzyme (Figure 4), and kinetic parameters could not be determined due to the low activity. These variants also had reduced propionyl-adenylate synthetase activity, with *k*_cat_ values reduced 4.5- to 21-fold (Supplemental Table S3). The *K_m_* values for propionate and ATP were not substantially affected in these variants except for the Tyr^525^Ala variant for which the *K_m_* value for propionate increased 6.0-fold and that for ATP decreased 14.7-fold (Supplemental Table S3).

To examine whether these alterations affected just the second step of the reaction in which CoA binding occurs or affected catalysis of the first step of the reaction as well, kinetic parameters were determined for the CoA-independent propionyl-adenylate synthetase activity of the enzyme. Except for the Tyr^525^Ala variant, the *K_m_* values for propionate and ATP showed ~2-fold or less change from the values observed for the unaltered enzyme although the *k*_cat_ values were ~2–10 fold decreased (Supplementary Table S3). These results suggest that although the first step of the reaction is affected, the impact is not enough to account for the near lack of acyl-CoA synthetase activity and that CoA binding and/or catalysis of the second step of the reaction are specifically affected.

**Figure 5:**
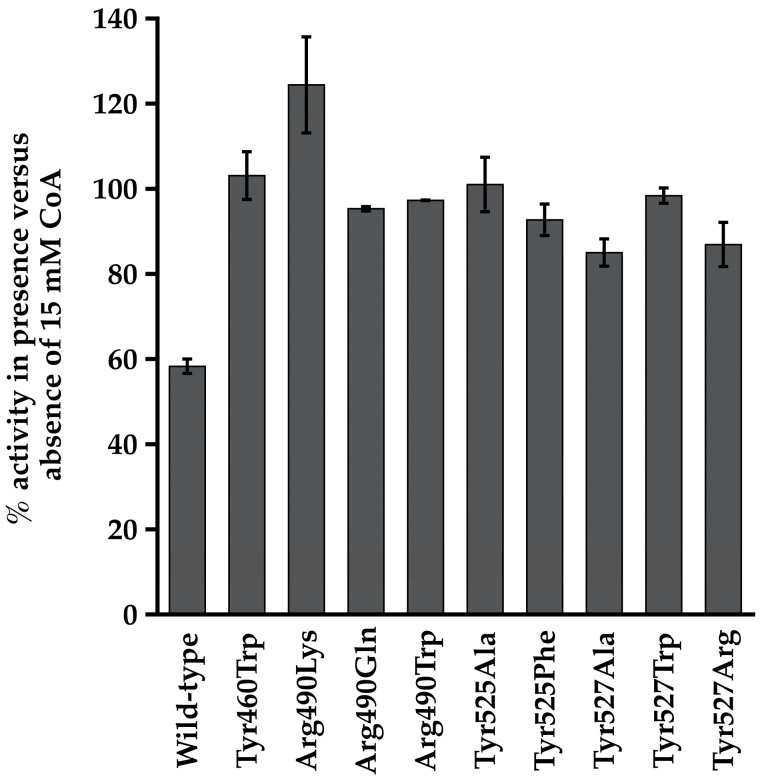
Effect of CoA on propionyl-adenylate synthetase activity of Macs_Ma_ wild-type and variants. Activities in the presence of 15 mM CoA were normalized as percentages relative to the specific activity observed for each enzyme in the absence of CoA. Reactions were performed in triplicate and values are the mean ± SD.

Because CoA inhibits the propionyl-adenylate synthetase activity of the wild-type enzyme [11], we examined the effect of a high concentration of CoA on this activity in the variants as a means for determining whether CoA can still bind even though the variants cannot catalyze the second step of the reaction. The presence of 15 mM CoA reduced activity of the wild-type enzyme by nearly 40% but had little to no inhibitory effect on activity of the variants (Figure 5). The presence of 15 mM CoA stimulated propionyl-adenylate synthetase activity of the Arg^490^Lys variant by nearly 25%. The reason for this is unknown and was not investigated further.

### 3.5. The Corresponding Lys Residues in Macs_Ma_ Do Not Play a Major Role in CoA Binding

**Figure 6:**
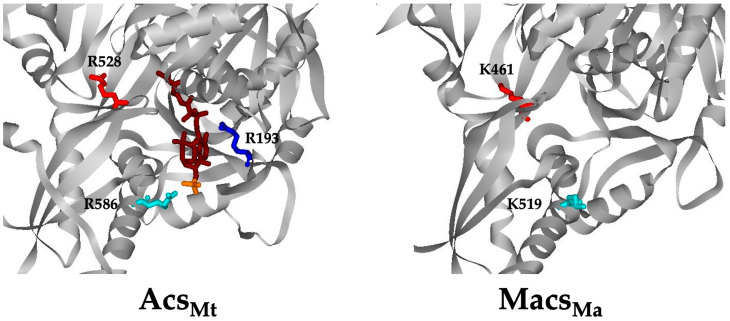
Positioning of corresponding residues Arg^528^/Lys^461^ (red) and Arg^586^/Lys^519^ (blue) in Acs_Mt_ (**left**) and Macs_Ma_ (**right**).

To provide further confirmation that CoA binding in Macs_Ma_ more closely resembles that in CBAL than Acs, we also examined the role of Lys^461^ and Lys^519^, which are positioned similarly to Arg^528^ and Arg^586^ of Acs_Mt_ (Figure 6). These Lys residues were individually altered to Arg and Ala and the purified variants were characterized. Kinetic parameters were determined using 2-methylbutyrate, the preferred substrate for the acyl-CoA synthetase activity of these enzyme variants. The Lys^461^Ala and Lys^461^Arg variants showed just 1.9-fold and 3.0-fold decrease, respectively, in the *K_m_* for CoA and a modest (less than 10-fold) decrease in the *K_m_* value for 2-methylbutyrate (Table 3). The *K_m_* values for 2-methylbutyrate and ATP were not substantially affected (Supplementary Table S2). These results suggest that Lys^461^ in Macs_Ma_ does not play a role similar to the corresponding Arg in Acs as there was little impact on CoA binding and catalysis.

The Lys^519^Arg alteration resulted in less than 2-fold change in the *K_m_* for any substrate or the turnover rate for the 2-methylbutyryl-CoA synthetase (Table 3 and Supplementary Table S2) or the propionyl-adenylate synthetase (Supplementary Table S3) activities. In contrast, the Lys^519^Ala variant had too little activity to determine kinetic parameters for either activity (Table 3 and Supplementary Tables S2 and S3).

## 4. Discussion

We have previously investigated substrate binding and catalysis in the short- and medium-chain acyl-CoA synthetases and identified residues important for acyl substrate binding in Acs and Macs and ATP binding in Acs. Here we examined CoA binding in Acs_Mt_ and Macs_Ma_.

Inspection of the Acs_Sc_ and Acs_Se_ structures [4,5] revealed two conformations for the enzyme. In the first step of the reaction, the C-terminal domain is positioned out and away from the active site but then swings in toward the N-terminal domain for catalysis of the second step of the reaction. Three Arg residues, Arg^191^, Arg^526^, and Arg^584^ (Arg^193^, Arg^528^, and Arg^586^ of Acs_Mt_, respectively) were proposed to play an important role in CoA binding and catalysis of the second step. Arg^191^ interacts with both the 5′-diphosphate and the 3′-diphosphate groups of CoA. As this residue is on the N-terminal domain and already present in the active site before domain alternation, it may play an important role in initial binding of CoA. Arg^584^ enters the active site after domain alternation to interact with the 3′-phosphate group, and Arg^526^, also on the C-terminal domain, was proposed to stabilize the thioester-forming conformation through interaction with the phosphate group of the acyl-adenylate intermediate [5]. Although this residue was not proposed to directly interact with CoA, it may influence CoA binding and catalysis by locking the enzyme in the thioester-forming conformation and thus encasing CoA in the active site.

We altered each of these Arg residues individually in Acs_Mt_ and assessed each variant’s kinetic abilities. All the variants were impaired in catalysis, with *k*_cat_ values reduced by 34 to 680-fold. The effect of these alterations on the *K_m_* for CoA varied, with alterations at Arg^193^ being the most detrimental and replacements at Arg^528^ and Arg^586^ having more variable effects. As might be expected, substitution with Ala at each position was the most deleterious, likely due to loss of both side chain charge and size. In fact, the Arg^193^Ala and Arg^528^Ala variants were not saturable for CoA or, surprisingly, for acetate.

Replacement of Arg^586^ had a lesser effect than replacement at Arg^193^, most likely because Arg^586^ only contacts CoA at the 3′-phosphate rather than at both the 5′-diphosphate and the 3′-phosphate as for Arg^193^. However, this single point of contact between Arg^586^ and CoA is important in CoA recognition and/or binding as shown by the fact that the Arg^586^ variants were unable to distinguish between CoA and 3′-dephosphoCoA.

Alteration of Arg^528^ increased the *K_m_* for CoA and decreased *k*_cat_ despite Arg^528^ appearing to contact the phosphate group of the acyl-adenylate intermediate rather than direct contact with CoA, thus stabilizing the thioester-forming conformation of the enzyme. Substitution at this position would then be expected to reduce the ability of the enzyme to maintain proper positioning of the acyl-adenylate intermediate in the active site, thus affecting catalysis and influencing the ability to bind CoA as well. Our results suggest these three Arg residues are essential for CoA binding and catalysis, directly or indirectly. These residues may also influence acetate binding in the first step of the reaction, perhaps through an inability to fully control domain alternation.

In contrast to our results, Reger et al. [6] reported that alteration of Arg^526^ and Arg^584^ of Acs_Se_ resulted in just a 2-fold decrease in catalysis. The *K_m_* for CoA increased for each of the variants, ranging from 4-fold for the Arg^526^Ala variant to 7 to 8-fold for the Arg^584^Ala and Arg^584^Glu variants [6]. However, no alterations were made at Arg^191^, the equivalent to Arg^193^ of Acs_Mt_. Reger et al. [6] determined the kinetic parameters for CoA and ATP using 20 mM acetate in the reaction mixture for all enzyme variants. Given that the wild-type enzyme has a reported *K_m_* for acetate of 6.05 mM, the kinetic constants for ATP and CoA may have been determined at subsaturating acetate concentrations. These inconsistencies between our results and those of Reger et al. [6] may thus reflect differences between the two Acs enzymes, which share only 49% sequence identity at the amino acid level, or the experimental conditions. Such differences among Acs enzymes were already noted for acyl substrate selection [9,10].

In the Macs_Ma_ structure, the enzyme was found to be in a similar conformation to that observed for Acs_Se_, as if poised for the second step of the reaction even in the absence of substrates [7]. Comparison of the CBAL [21] and Acs_Se_ structures [5] in the conformation for the second step of the reaction revealed that the binding pockets for CoA nucleotide moiety in these enzymes are significantly different [21], with more interactions with the N-terminal domain in Acs but with the C-terminal domain for CBAL. Superposition of the Macs_Ma_ structure with the CBAL and Acs_Se_ structures indicates that the CoA binding site more closely resembles that of CBAL [7]. The recent structure of a 2-hydroxyisobutyric acid CoA ligase shows CoA binding in a substantially bent conformation in the thioester conformation [28]. This contrasts with the more stretched conformations of CoA observed in the thioester forming conformations of CBAL, Macs, and Acs.

We investigated five residues in Macs_Ma_ predicted to interact with CoA based on comparison with the CBAL structure. Tyr^460^, Arg^590^, Tyr^525^, or Tyr^527^ variants displayed greatly reduced 2-methylbutyryl-CoA synthetase activity, and propionyl-adenylate synthetase activity was also reduced but to a much lesser extent. Alteration at Gly^459^, which is strictly conserved in the acyl-adenylate-forming superfamily [7] reduced the turnover rate for both enzymatic activities but did not substantially affect the *K_m_* values for substrates.

In order to examine whether the alterations in the putative CoA binding pocket residues affected just catalysis or also affect CoA binding, we took advantage of the fact that CoA inhibits the propionyl-adenylate synthetase activity of Macs_Ma_ [11]. In each case, the variant showed less inhibition of the propionyl-adenylate synthetase activity by CoA than for the wild-type enzyme, suggesting that CoA cannot bind as well and supporting that these four residues play a key role in CoA binding as well as catalysis by Macs_Ma_.

Macs_Ma_ lacks each of the three Arg residues investigated in Acs_Mt_. However, Arg^528^ and Arg^586^ of Acs_Mt_ are replaced by Lys residues at the corresponding positions (residues 461 and 519) in Macs_Ma_. Structurally, although these residues are in the vicinity of the predicted CoA binding pocket of Macs_Ma_, they are more remote from CoA than the Arg residues of Acs. Our kinetics results for Macs_Ma_ variants altered at these Lys residues suggest that Lys^461^ does not play a role in CoA binding or catalysis. Although Lys^519^ may play some role, maintenance of the positive charge at this position is sufficient. Alterations at these positions (with the exception of a Lys^519^Ala alteration) resulted in only mild reductions in *k*_cat_ (5-fold or less) for either the acyl-CoA synthetase activity or the propionyl-adenylate synthetase activity. Per residue binding free energy decomposition had previously identified Lys^461^ as a residue important in 2-methyl butyrate binding and catalysis [29]. Our alteration of Lys^461^ to alanine and arginine had only 7.5-fold and 9-fold reduction on *K_m_*, respectively.

Overall, although Acs and Macs have similarities in active site architecture for substrate binding and catalysis of the first step of the reaction, our results strongly suggest that the active site architecture for CoA binding and catalysis of the second step has diverged greatly. Although structural comparison between Acs_Se_ and Macs_Ma_ revealed distinct differences in the CoA binding pocket [7], it appears that electrostatic interaction with the 3′-phosphate group of CoA is important for both enzymes; however, this interaction occurs with disparate residues in each enzyme. Differences in acyl substrate binding sites among acyl-CoA synthetase family members is not surprising as the enzymes must accommodate substrates of different lengths that may be branched or unbranched. However, the diversity in CoA binding sites among family members was unexpected.

## Figures and Tables

**Table 1:** Kinetics parameters for Acs_Mt_ wild-type and variant enzymes.

Enzyme	*K_m_* CoA (mM)	*k*_cat_ (sec^−1^)	*k*_cat_/*K_m_* (sec^−1^ mM^−1^)
Wild-type ^a^	0.19 ± 0.003	81.6 ± 0.7	423.7 ± 4.7
Arg^193^Ala	Unsaturable ^b^		
Arg^193^Lys	3.6 ± 0.37	0.51 ± 0.01	0.14 ± 0.01
Arg^193^Gln	7.8 ± 0.40	0.28 ± 0.004	0.036 ± 0.002
Arg^528^Ala	Unsaturable ^b^		
Arg^528^Lys	0.45 ± 0.03	0.25 ± 0.01	0.54 ± 0.02
Arg^528^Gln	1.67 ± 0.05	0.63 ± 0.04	0.38 ± 0.06
Arg^586^Ala	1.19 ± 0.08	2.13 ± 0.09	1.80 ± 0.06
Arg^586^Lys	0.33 ± 0.013	2.39 ± 0.016	7.34 ± 0.24
Arg^586^Gln	0.28 ± 0.009	0.12 ± 0.003	0.45 ± 0.028

^a^ Values are taken from [9]. ^b^ The enzyme was not saturable for CoA at concentrations up to 25 mM and kinetic parameters could not be determined.

**Table 2:** Discrimination between CoA and 3′-dephospho CoA (deCoA) for wild-type Acs_Mt_ and the Arg^586^ variants.

Enzyme	Substrate	*K_m_* (mM)	*k*_cat_ (sec^−1^)	*k*_cat_/*K_m_* (sec^−1^ mM^−1^)	(*k*_cat_/*K_m_* CoA)/ (*k*_cat_/*K_m_* deCoA)
Wild-type	CoA	0.19 ± 0.003	81.6 ± 0.7	423.7 ± 4.7	26.5
	deCoA	2.16 ± 0.50	34.5 ± 2.9	16.0 ± 2.5	
Arg^586^Ala	CoA	1.19 ± 0.08	2.13 ± 0.09	1.80 ± 0.06	2.2
	deCoA	2.05 ± 0.40	1.66 ± 0.11	0.81 ± 0.11	
Arg^586^Lys	CoA	0.33 ± 0.01	2.39 ± 0.02	7.34 ± 0.20	3.7
	deCoA	0.86 ± 0.04	1.72 ± 0.01	2.01 ± 0.09	

**Table 3:** Kinetic parameters for the 2-methylbutyryl-CoA synthetase activity for wild-type Macs_Ma_ and the Gly^459^, Lys^461^, and Lys^519^ variants.

Enzyme	*k*_cat_ (sec^−1^)	*K_m_* CoA (mM)
Wild-type	2.15 ± 0.10	4.09 ± 0.45
Gly^459^Ala	0.41 ± 0.01	2.09 ± 0.06
Lys^461^Ala	0.55 ± 0.03	2.19 ± 0.18
Lys^461^Arg	0.46 ± 0.10	1.38 ± 0.14
Lys^519^Ala	*	*
Lys^519^Arg	1.07 ± 0.02	7.18 ± 0.29

* Activity was too low for determination of kinetic parameters.

## Data Availability

All data relevant to this study are reported within.

## Supplementary Materials

The following supporting information can be downloaded at: https://www.mdpi.com/article/10.3390/life13081643/s1, Table S1: *K_m_* values for acetate and ATP for Acs_Mt_ wild-type and variant enzymes; Table S2: *K_m_* values for 2-methylbutyrate and ATP for wild-type Macs_Ma_ and the Lys^461^, Lys^519^, and Gly^459^ variants; Table S3: Kinetic parameters for the propionyl-adenylate synthetase activity of wild-type Macs_Ma_ and the Lys^461^, Lys^519^, Gly^459^, Tyr^460^, Tyr^525^, Tyr^527^, and Arg^490^ variants.
