# Supplementary material for: The Roles of Coenzyme A Binding Pocket Residues in Short and Medium Chain Acyl-CoA Synthetases
Source: Life (Basel). 2023 Jul 28;13(8):1643. doi: 10.3390/life13081643 (PMC10455477; doi:10.3390/life13081643)
Supplement: Supplementary file 1 [file life-13-01643-s001.zip › life-2464737-supplementary.pdf]

Supplemental Table S1. K_m values for acetate and ATP for Acs_{Mt} wild-type and variant enzymes

Enzyme	K_m Acetate (mM)[a]	K_m ATP (mM)[a]
Wild-type[a]	3.5 ± 0.1	3.3 ± 0.2
Arg^{193}Ala	Unsaturable[b]	6.1 ± 0.2[c]
Arg^{193}Lys	1.6 ± 0.01	1.8 ± 0.12
Arg^{193}Gln	1.1 ± 0.08	0.7 ± 0.02
Arg^{528}Ala	Unsaturable[b]	1.7 ± 0.31[c]
Arg^{528}Lys	1.4 ± 0.24	1.5 ± 0.13
Arg^{528}Gln	2.0 ± 0.16	1.8 ± 0.23
Arg^{586}Ala	1.0 ± 0.05	2.3 ± 0.11
Arg^{586}Lys	3.1 ± 0.01	2.6 ± 0.3
Arg^{586}Gln	Unsaturable[b]	3.6 ± 0.4[c]

[a] Values are taken from (9).
[b] The enzyme was not saturable for acetate at concentrations up to 800 mM.
[c] The K_m for ATP was determined in the presence of 100 mM acetate and 20 mM CoA.

Supplemental Table S2. K_m values for 2-methylbutyrate and ATP for wild-type $Macs_{Ma}$ and the Lys^{461}, Lys^{519}, and Gly^{459} variants

Enzyme	K_m 2-MB (mM)	K_m ATP (mM)
Wild-type	8.92 ± 0.51	4.21 ± 0.11
Lys^{461}Ala	1.18 ± 0.09	1.64 ± 0.09
Lys^{461}Arg	0.96 ± 0.06	1.28 ± 0.06
Lys^{519}Ala	*	*
Lys^{519}Arg	7.96 ± 0.56	4.63 ± 0.11
Gly^{459}Ala	3.07 ± 0.30	3.31 ± 0.05

*Activity was too low for determination of kinetic parameters

Supplemental Table S3. Kinetic parameters for the propionyl-adenylate synthetase activity of wild-type $Macs_{Ma}$ and the Lys^{461}, Lys^{519}, Gly^{459}, Tyr^{460}, Tyr^{525}, Tyr^{527}, and Arg^{490} variants

Enzyme	k_{cat} (sec^{-1})	K_m propionate (mM)	K_m ATP (mM)
Wild-type	10.67 ± 1.34	29.10 ± 0.30	6.90 ± 0.75
Lys^{461}Ala	4.22 ± 0.04	91.49 ± 1.00	10.09 ± 0.11
Lys^{461}Arg	6.73 ± 0.15	33.47 ± 3.79	4.99 ± 0.46
Lys^{519}Ala	*	*	*
Lys^{519}Arg	2.64 ± 0.12	41.60 ± 1.80	6.47 ± 0.49
Gly^{459}Ala	2.30 ± 0.06	86.31 ± 3.30	6.15 ± 0.31
Tyr^{460}Trp	0.56 ± 0.001	29.60 ± 3.50	4.23 ± 0.16
Tyr^{525}Ala	0.58 ± 0.02	175.35 ± 8.20	0.47 ± 0.02

$Tyr^{525}Phe$	1.27 ± 0.02	60.39 ± 4.98	5.17 ± 0.10
$Tyr^{527}Ala$	2.37 ± 0.10	49.28 ± 1.93	3.24 ± 0.07
$Tyr^{527}Trp$	1.47 ± 0.06	33.07 ± 0.36	4.28 ± 0.14
$Tyr^{527}Arg$	0.51 ± 0.004	25.81 ± 0.47	3.05 ± 0.11
$Arg^{490}Lys$	0.73 ± 0.004	50.17 ± 0.88	5.78 ± 0.29
$Arg^{490}Gln$	1.60 ± 0.05	45.39 ± 0.58	5.43 ± 0.56
$Arg^{490}Trp$	0.81 ± 0.01	62.64 ± 2.20	4.96 ± 0.02

*Activity was too low for determination of kinetic parameters
